# Interleukin‐37 inhibits osteoclastogenesis and alleviates inflammatory bone destruction

**DOI:** 10.1002/jcp.27526

**Published:** 2018-11-10

**Authors:** Ruohui Tang, Jin Yi, Jing Yang, Yueqi Chen, Wei Luo, Shiwu Dong, Jun Fei

**Affiliations:** ^1^ Center of Trauma of Daping Hospital, Third Military Medical University Chongqing China; ^2^ Department of Biomedical Materials Science, School of Biomedical Engineering, Third Military Medical University (Army Medical University) Chongqing China; ^3^ Department of Osteological Guizhou Province People’s Hospital Guiyang China; ^4^ State Key Laboratory of Trauma, Burns and Combined Injury, Third Military Medical University Chongqing China

**Keywords:** inflammatory bone destruction, interleukin‐37, lipopolysaccharide, nuclear factor‐κB signaling, osteoclast differentiation

## Abstract

Excessive osteoclast formation is one of the important pathological features of inflammatory bone destruction. Interleukin‐37 (IL‐37) is an anti‐inflammatory agent that is present throughout the body, but it displays low physiological retention. In our study, high levels of the IL‐37 protein were detected in clinical specimens from patients with bone infections. However, the impact of IL‐37 on osteoclast formation remains unclear. Next, IL‐37 alleviated the inflammatory bone destruction in the mouse in vivo. We used receptor activator of nuclear factor‐κB ligand and lipopolysaccharide to trigger osteoclastogenesis under physiological and pathological conditions to observe the role of IL‐37 in this process and explore the potential mechanism of this phenomenon. In both induction models, IL‐37 exerted inhibitory effects on osteoclast differentiation and bone resorption. Furthermore, IL‐37 decreased the phosphorylation of inhibitor of κBα and p65 and the expression of nuclear factor of activated T cells c1, while the dimerization inhibitor of myeloid differentiation factor 88 reversed the effects. These data provide evidence that IL‐37 modulates osteoclastogenesis and a theoretical basis for the clinical application of IL‐37 as a treatment for bone loss–related diseases.

## 1 INTRODUCTION

Bone and joint infections, including orthopedic implant‐associated infections, osteoarthritis, and osteomyelitis, are associated with severe bone destruction. A recent survey of 537,575 cases of revision total hip arthroplasty (RTHA) and revision total knee arthroplasty (RTKA) in the United States revealed that bone infection was responsible for RTHA (15.4%) and RTKA (25%; Birt, Anderson, Bruce Toby, & Wang, 2017). The symptoms of bone infection include pain and varying degrees of bone destruction. Severe inflammatory bone destruction is closely related to disorders of bone metabolism. The balance between the functional of various cells involved in bone metabolism is destroyed in bone infection. In addition to surgical debridement and antibiotic treatments for bone infections, an immunotherapy targeting functional cells in the skeletal system has attracted attention. Bone homeostasis is impacted by immunocytes and their secreted products by influencing the functions of osteocytes, osteoblasts, and osteoclasts (Mbalaviele, Novack, Schett, & Teitelbaum, 2017). Osteoclasts are responsible for bone resorption in the body. Among the many types of proinflammatory cytokines, the most typical proteins are interleukin 1β (IL‐1β), IL‐6, tumor necrosis factor‐α (TNF‐α), and IL‐17 (Wojdasiewicz, Poniatowski, & Szukiewicz, 2014). IL‐17 has been widely recognized in autoimmune diseases that are involved in the local inflammatory process by producing proinﬂammatory cytokines (Takayanagi, 2009). During the inflammatory response, Th17 cells synthesize and secrete IL‐17 and receptor activator of nuclear factor‐κB ligand (RANKL), both of which promote osteoclast formation (Kotake et al., 1999). RANKL is a member of the TNF superfamily, which has been widely shown to be required for osteoclast differentiation.

As a component of the IL‐1 family, IL‐37 was identified as a natural inhibitor of immune responses (Nold et al., 2010). Five subtypes of IL‐37, IL‐37a to IL‐37e, are produced by alternative splicing. Among these variants, IL‐37b is the largest of the five isoforms, with the greatest number of exons and biological function (Dinarello et al., 2016). IL‐37b is expressed in many tissues and different cell types, such as the bone marrow, liver, lymph nodes, monocytes, dendritic cells, and endothelial cells (Tete et al., 2012). IL‐37 silencing in human peripheral blood monocytes significantly increases the levels of inﬂammatory cytokines in vitro (Nold et al., 2010). Researchers have used several animal models to study this critical anti‐inflammatory factor. The recombinant IL‐37 protein has been shown to function in wild‐type mouse models of diseases, such as ischemic injury, hepatitis, and rheumatoid arthritis (Dinarello et al., 2016; Ding et al., 2017; Sakai et al., 2012), but fewer studies have reported an association of IL‐37 with bone infections. In the current study, we examined the levels of the IL‐37 protein clinical specimens of infected bones. Additional experiments were performed to evaluate the impact of IL‐37 on lipopolysaccharide (LPS)‐mediated inflammatory bone destruction in vivo. Theoretically, IL‐37 indirectly affects osteoclast differentiation by modulating the expression of proinflammatory factors. However, the direct effect of IL‐37 in this process remains unclear. We constructed two models of physiological and LPS‐simulated inflammation to explore the link between IL‐37 and osteoclastogenesis.

In this study, the levels of the IL‐37 protein were detected in specimens from patients with bone infections, strongly confirming that this protein may be involved in the fracture repair process. The efficacy of the recombinant IL‐37 protein at preserving bone mass in the LPS‐mediated inflammatory bone destruction model was verified. According to subsequent in vitro experiments, IL‐37 inhibits RANKL‐mediated or LPS‐mediated osteoclast formation in vitro, which may explain how the protein protects bone mass, while the nuclear factor‐κB (NF‐κB) signaling pathway is involved in the regulatory process. The identification of the negative regulators of IL‐37 function in osteoclastogenesis may provide a basis for improving treatments for bone infections.

## 2 MATERIALS AND METHODS

### 2.1 Reagents

Recombinant human IL‐37b/IL‐1F7b protein (7585‐IL‐025/CF), recombinant murine RANKL (462‐TEC‐010/CF), and macrophage colony stimulating factor (M‐CSF; 416‐ML‐010) were from R&D Systems (Minneapolis, MN). Antibodies against p65, p‐p65, inhibitor of κBα (IκBα), p‐IκBα, and glyceraldehyde 3‐phosphate dehydrogenase (GAPDH) were from Cell Signaling (Danvers, MA). Antibodies against nuclear factor of activated T cells 1 (NFATc1; sc‐13033), myeloid differentiation factor 88 (MyD88; sc‐74532), and single Ig IL‐1‐related receptor (SIGIRR; sc‐515818) were from Santa Cruz Biotechnology (Santa Cruz, CA). Antibody against IL‐37 (ab‐57187) and RANKL (ab‐9957) were obtained from Abcam (Cambridge, MA). Osteo Assay Surface was from Corning (Corning, NY). The cell counting kit‐8 (CCK‐8) was purchased from Dojindo Molecular Technologies (Dojindo, Japan). The tartrate‐resistant acid phosphatase (TRAP) stain kit was from Sigma‐Aldrich (St. Louis, MO). The Focal Adhesion and Actin Cytoskeleton Staining Kit was obtained from Millipore (Darmstadt, Germany). MyD88 pharmacologic inhibitor (ST 2825) was purchased from Medchemexpress (Princeton, NJ). LPSs from *Escherichia coli O55:B5* were purchased from Sigma‐Aldrich. Coimmunoprecipitation (Co‐IP) Kit (Pierce; 88804) were purchased from Thermo Fisher Scientific Inc., (Waltham, MA).

### 2.2 Clinical specimens

Clinical specimens were collected from patients at Daping Hospital (Chongqing, China). The study followed the law and was approved by the Daping Hospital Clinical Ethics Committee. All specimens were collected with the informed consent of the patients. Bone and adjacent tissue specimens were removed from six patients with traumatic osteomyelitis‐infected or noninfectious closed fractures. Samples were fixed with 4% paraformaldehyde (PFA) for 24 hr and processed into paraffin sections with a thickness of 5 μm for hematoxylin and eosin (H&E) staining. Immunohistochemistry for IL‐37 was performed on sections using a primary antibody against IL‐37 (1:200). Stained sections were observed under a microscope (DMI 6000B; Leica, Wetzlar, Germany).

### 2.3 Cytotoxicity and apoptosis assays

The cell counting kit‐8 (CCK‐8) assay was performed to measure cell proliferation, according to the manufacturer’s instructions. RAW264.7 cells were seeded onto 96‐well plates at a density of 0.3 × 10^4^ cells/well. Cells were treated with different concentrations of IL‐37b in the presence of RANKL (50 ng/ml) and M‐CSF (50 ng/ml) for 48 and 72 hr, respectively. After completing the abovementioned procedures, 10 μl of CCK‐8 working solution was added to each well and the plate was incubated at 37°C for 2 hr. The absorbance was measured at 450 nm using a spectrophotometer (Synergy H4; BioTek Instruments, Winooski, VT). We measured the apoptosis rate of cultured cells using flow cytometry (BD, Triangle, NC). Precursor cells were divided into a RANKL pretreatment group and nonpretreatment group, respectively, and were then stimulated with 100 ng/ml LPS and 50 ng/ml M‐CSF for 72 hr. Annexin V‐FITC/PI staining was performed to detect the apoptosis of cultured cells. This procedure was performed according to the manufacturer’s instructions (Life Technologies, Carlsbad, CA).

### 2.4 Cell culture and osteoclast differentiation analyses

RAW264.7 cells were purchased from the American Type Culture Collection (Rockville, MD). Before the induction experiment, mouse osteoclast precursor RAW264.7 cells were incubated in a complete medium supplemented with 10% fetal bovine serum ( Life Technologies), 1% penicillin and streptomycin (Thermo Fisher Scientific) at 37°C for 24 hr. Precursor cells were seeded onto a plate in the medium containing RANKL (50 ng/ml) and M‐CSF (50 ng/ml) and stimulated with or without IL‐37b (50–200 ng/ml) for 72 hr. Culture media were refreshed for 2 days. Isolated bone marrow macrophages from C57BL/6 mice were obtained using previously reported methods (Dou et al., 2016) and were cultured with M‐CSF (50 ng/ml) and RANKL (50 ng/ml) for 5 days.

Cells were washed twice with phosphate‐buffered saline (PBS) before an incubation with 4% paraformaldehyde for 20 min. Then, we evaluated the process of osteoclast formation by performing TRAP staining and the number of TRAP‐positive multinucleated cells with three or more nuclei under an optical microscope (DMI 6000B; Leica). In addition, RAW264.7 cells were pretreated with RANKL and M‐CSF for 24 hr and washed twice before being stimulated with M‐CSF (50 ng/ml) or LPS (100 ng/ml) in the presence or absence of IL‐37b (200 ng/ml) for 72 hr (Shinohara et al., 2015). After culture, cells were stained with TRAP and focal adhesion and actin cytoskeleton (FAK). Next, we investigated the effect of IL‐37b on osteoclast differentiation under LPS‐induced inflammatory conditions.

### 2.5 Bone resorption assay in vitro

To verify the effect of IL‐37b on osteoclast bone resorption in vitro, preosteoclasts were transferred onto 96‐well plates coated with hydroxyapatite (Osteo Assay Surface; Corning, NY). Cells were cultured with RANKL (50 ng/ml) and M‐CSF (50 ng/ml) for 5 days in the presence or absence of IL‐37b at 200 ng/ml. We evaluated pit formation using a method described in a previous report (Dou et al., 2016), and the absorption area was analyzed using the ImageJ software (NIH, Bethesda, MD).

### 2.6 RNA isolation and quantitative real‐time polymerase chain reaction (qRT‐PCR)

RAW264.7 cells were stimulated with RANKL (50 ng/ml) and M‐CSF (50 ng/ml) for 24 or 72 hr. The experimental group was additionally supplemented with 100 or 200 ng/ml IL‐37b. We extracted the total RNA from cultured cells using TRIzol reagent (Life Technologies). The RNA was synthesized into complementary DNAs using a reverse transcription kit  (Takara Bio, Shiga, Japan). qRT‐PCR was performed using SYBR Premix Ex Taq II (Takara Bio Inc.) in a PCR detection system (Bio‐Rad, Hercules, CA), and the primer sequences are shown in Table 1. The expression of the target gene was normalized to the levels of the GAPDH messenger RNA (mRNA).

**Table 1:** Primer sequences for qRT‐PCR

Genes	Forward	Reverse
NFATc1	5’‐CCCGTCACATTCTGGTCCAT‐3’	5’‐CAAGTAACCGTGTAGCTGCACAA‐3’
c‐Fos	5’‐CGGGTTTCAACGCCGACTA‐3’	5’‐TTGGCACTAGAGACGGACAGA‐3’
MMP9	5’‐CTGGACAGCCAGACACTAAAG‐3’	5’‐CTCGCGGCAAGTCTTCAGAG‐3’
CtsK	5’‐GAAGAAGACTCACCAGAAGCAG‐3’	5’‐TCCAGGTTATGGGCAGAGATT‐3’
Ctr	5’‐CGCATCCGCTTGAATGTG‐3’	5’‐TC TGTCTTTCCCCAGGAAATGA‐3’
DC‐STAMP	5’‐CTAGCTGGCTGGACTTCATCC‐3’	5’‐TCATGCTGTCTAGGAGACCTC‐3’
PU.1	5’‐GATGGAGAAGCTGATGGCTTGG‐3’	5’‐TTCTTCACCTCGCCTGTCTTGC‐3’
IL‐1β	5’‐CTCAACTGTGAAATGCCACC‐3’	5’‐TGTCCTCATCCTGGAAGGT‐3’
IL‐6	5’‐TGGGAAATCGTGGAAATGAGA‐3’`	5’‐ACTCTGGCTTTGTCTTTCTTGT‐3’
TNF‐α	5’‐AGGCGGTGCTTGTTCCTCA‐3’	5’‐AGGCGAGAAGATGATCTGACTGCC‐3’
GAPDH	5’‐AAA TGGTGAAGGTCGGTGTG‐3’	5’‐TGAAGGGGTCGTTGATGG‐3’

*Note*. Ctr: calcitonin receptor ; CtsK: cathepsin K; DC‐STAMP: dendritic cell‐specific transmembrane protein; GAPDH: glyceraldehyde 3‐phosphate dehydrogenase; IL‐1β: interleukin‐1β; MMP9: matrix metalloproteinase‐9; NFATc1: nuclear factor of activated T cells 1; qRT‐PCR, quantitative real‐time polymerase chain reaction; TNF‐α, tumor necrosis factor‐α.

### 2.7 Western blot and Co‐IP analyses

We treated RAW264.7 cells that had been cultured with RANKL and M‐CSF with 200 ng/ml IL‐37b, and then, we extracted the total proteins in radioimmunoprecipitation assay (RIPA) buffer containing phosphatase inhibitors and protease inhibitors. For samples obtained during surgery, specimens were repeatedly crushed in liquid nitrogen, resulting in the production of a supernatant containing the protein sample. Lysates were separated on 10% sodium dodecyl sulfate polyacrylamide gel electrophoresis gels and transferred to the polyvinylidene difluoride membranes. All membranes were blocked in a TBST solution (tris‐buffered saline [TBS] containing 0.1% Tween) containing 5% nonfat milk for 3 hr before an overnight incubation with primary antibodies (NFATc1, GAPDH, IκBα, p‐IκBα [phospho‐S32], p‐p65 [phospho‐S536], p65, or IL‐37) at 4°C. After the completion of this process, membranes were washed three times with TBST and immersed in secondary antibody at room temperature for 1.5 hr. For this experiment, GAPDH expression served as an internal control.

For the Co‐IP analysis, RAW264.7 cells were treated with M‐CSF (50 ng/ml), RANKL (50 ng/ml), and IL‐37b (200 ng/ml) for 48 hr. Cells were lysed in RIPA buffer after washes with PBS. The supernatant was incubated with a SIGIRR antibody (1:50) or mouse IgG overnight at 4°C, followed by an incubation with protein A/G magnetic beads for 4 hr at 4°C. The complexes were washed with PBS and subjected to western blot analysis using antibodies against SIGIRR (1:2,000), MyD88 (1:2,000), and GAPDH.

### 2.8 Immunofluorescence staining

Precursor cells were incubated in the presence or absence of 200 ng/ml IL‐37b for 1 hr before treatment with RANKL for an additional 30 min. Cells were fixed with 4% PFA for 20 min, and permeabilization and blocking was performed before the cells were incubated with the primary antibody at 4°C. Cells were washed three times with PBS and then incubated with a Cy3‐conjugated secondary antibody and 4’,6‐diamidino‐2‐phenylindole (DAPI) in the dark. The colocalization of the p65 protein and nucleus was consistent with the previous reports (Fuchsová, Novák, Kafková, & Hozák, 2002), and the gray value was analyzed using the ImageJ software (NIH, Bethesda, MD). For focal adhesion and actin cytoskeleton stainingthe blocked cells were stained with antivinculin antibody (1:300) and tetramethylrhodamine‐5‐isothiocyanate‐conjugated phalloidin (1:300), which labels F‐actin, and then incubated with an Alexa Fluor 488‐conjugated secondary antibody. The nuclei were stained with DAPI before microscopic observations.

### 2.9 Inflammatory bone destruction model and radiographic analysis

Six‐week‐old C57BL/6 female mice were obtained from the animal center of the Third Military Medical University. The whole procedure was performed according to the guidelines of the Animal Care and Use Committee of the Third Military Medical University. Twelve mice were randomly allocated to three groups (*n* = 4 in each group): the control group (PBS‐treated), an LPS‐treated group, and the IL‐37b‐treated group, which received LPS and IL‐37b (4 μg/day) treatments. All animals were anesthetized by an intraperitoneal injection of 4% chloral hydrate (5 μl/g) to reduce the level of suffering. As described in the literature (Chiang, Kyritsis, Graves, & Amar, 1999), the mice were subcutaneously injected with LPS (100 μg/day) for 7 days before sacrifice to induce inflammatory bone destruction. The calvarial bones were fixed, decalcified, and cut into 5‐μm‐thick sections for H&E staining and TRAP staining.

For the micro‐computed tomography (micro‐CT) analysis, the femur tissues were scanned with viva CT 40 instrument (Scanco Medical, Brüttisellen, Switzerland) and the bone volume per tissue volume was calculated as described in a previous study (Dou et al., 2016). The scan of the trabecular bone was performed from the growth plate. The 3D images and bone metrology analysis were obtained by standard methods (Scanco Medical; CT Evaluation Program, V5.0A).

### 2.10 Statistics

All procedures were repeated at least three times to verify the results. The analysis was performed using GraphPad Prism 7(GraphPad Software, La Jolla, CA). The data were represented as the mean ± SD, and the significant differences in data among groups were compared by one‐way analysis of variance. Values of *P < 0.05 and **P < 0.01 were considered to be statistically significant.

## 3 RESULTS

### 3.1 High levels of the IL‐37 protein accumulate in bone‐infected lesions

**Figure 1:**
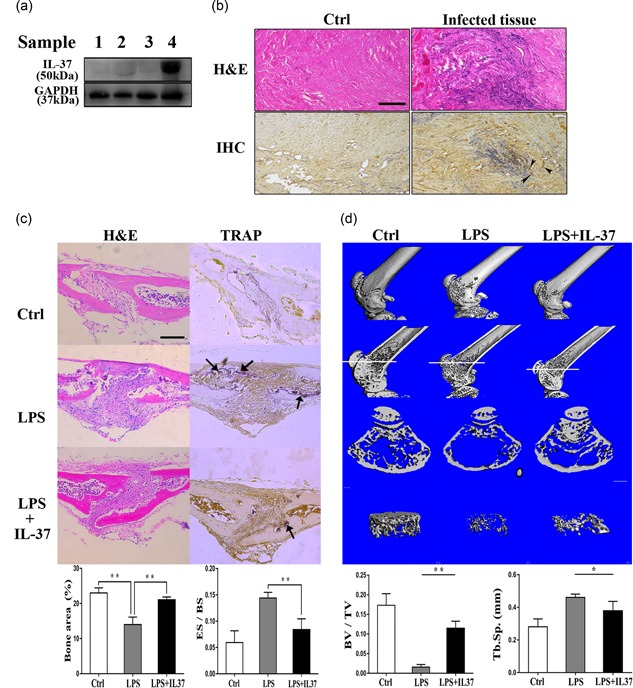
The level of the IL‐37 protein was detected in clinical specimens from patients with bone infections. The exogenous administration of IL‐37 attenuated LPS‐induced inflammatory bone destruction in mice. (a) Four groups of samples were obtained noninfectious fractures and traumatic osteomyelitis‐infected samples. Samples (Birt et al., 2017; Mbalaviele et al., 2017; Takayanagi, 2009; Wojdasiewicz et al., 2014) were extracted from normal cancellous bone, infected cancellous bone, normal connective tissue, and infected connective tissue, respectively. Levels of IL‐37 (IL‐37b) and GAPDH were detected by immunoblotting. (b) Representative images of H&E staining and immunohistochemical staining. Arrows indicate IL‐37‐positive cells. Scale bar = 200 μm. (c) TRAP staining, immunohistochemical staining, and H&E staining in sections of calvarial bones from 6‐week‐old C57BL/6 mice treated with PBS, LPS, or LPS + IL‐37b. Arrows indicate TRAP‐positive cells. Scale bar = 400 μm; *N* = 4. (d) The 3D images, images of longitudinal sections, images of cross‐sections, and trabecular images were reconstructed at the distal femur. Quantiﬁcation of Tb.Sp. and BV/TV in each group. Scale bar = 1 mm; *N* = 4. BV/TV: bone volume per tissue volume; Ctrl: control; ES/BS: eroded surface per bone surface; GAPDH: glyceraldehyde 3‐phosphate dehydrogenase; H&E: hematoxylin and eosin; IL‐37: interleukin‐37; LPS: lipopolysaccharide; PBS: phosphate‐buffered saline; Tb.Sp.: trabecular spacing; TRAP: tartrate‐resistant acid phosphatase [Color figure can be viewed at wileyonlinelibrary.com]

Increased IL‐37 levels have been confirmed in numerous inflammatory and allergic diseases (Quirk & Agrawal, 2014). To investigate whether inflammation caused the local production of IL‐37, we examined the expression of the IL‐37 protein in bones and surrounding tissues. Immunoblotting was also performed to evaluate the IL‐37 expression in paraffin‐embedded sections from fractures and the surrounding nonskeletal tissues. As shown in Figure 1a, IL‐37 was expressed at high levels in the infected connective tissues near the fracture. In images of H&E staining, we observed significant inflammatory cell infiltration in sections from patients with bone infections, and we observed a locally high expression of IL‐37 at approximately the same location using immunohistochemistry (Figure 1b). On the basis of these findings, the effects of elevated levels of the IL‐37 protein on bone remodeling became the focus of our study.

### 3.2 IL‐37 prevents inflammatory bone destruction in vivo

Although controversy exists regarding whether LPS may directly stimulate osteoclast differentiation, LPS causes inflammation, shock, and bone destruction in vivo. Therefore, we evaluated the potential correlation between the effect of IL‐37 on bone loss and inflammation in the LPS‐treated model. In fact, when the mice were subcutaneously injected with LPS, we observed a significant decrease in their activity and viability due to endotoxemia. The information we obtained from the sections of a 7‐day treatment group showed that IL‐37b effectively relieved LPS‐induced localized inflammatory responses and severe bone destruction. In addition, TRAP staining revealed a significant reduction in the number of osteoclasts in the IL‐37b‐treated group (Figure 1c). Micro‐CT was performed using isolated femurs. The bone metrology analysis revealed a significant increase in the bone volume per tissue volume and a decreased trabecular spacing following the application of IL‐37b to the mouse model of LPS‐induced bone loss (Figure 1d). Thus, IL‐37b to be involved in the mechanism regulating inflammatory bone loss in vivo. Therefore, we further verified whether the protective effect of IL‐37b on inflammatory bone destruction was achieved by regulating osteoclast differentiation in vitro.

### 3.3 IL‐37 suppresses RANKL‐mediated osteoclastogenesis and inhibits the bone‐resorbing activity of osteoclasts

**Figure 2:**
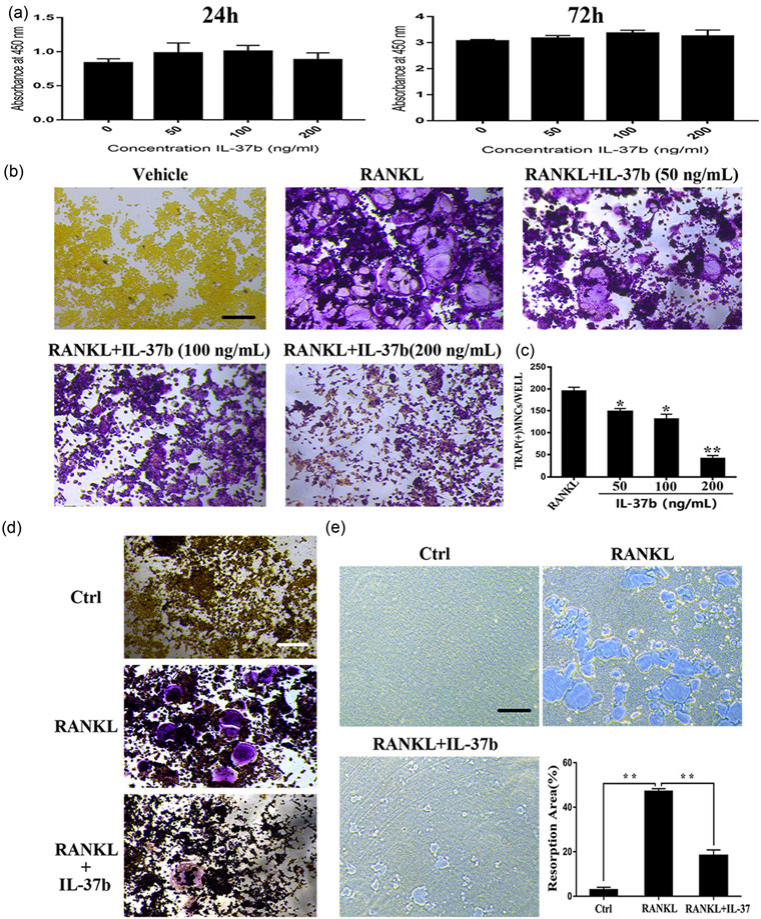
IL‐37b suppressed RANKL‐induced osteoclast differentiation without inducing cytotoxicity. (a) RAW264.7 cells were seeded onto 96‐well plates and incubated for 24 or 72 hr with different concentrations of IL‐37b. The CCK‐8 assay was used to detect cell proliferation. (b) Precursor cells were cultured with various concentrations of IL‐37b followed by M‐CSF (50 ng/ml) and RANKL (50 ng/ml) stimulation for 72 hr. These cells were simultaneously exposed to all of these factors. The steps for TRAP staining are described in the methods. Scale bar = 200 μm. (c) TRAP‐positive multinucleated cells ( ≥ 3 nuclei) were identified as osteoclasts and were counted. (d) BMMs were treated with M‐CSF (50 ng/ml), RANKL (50 ng/ml), and IL‐37 (200 ng/ml) for 5 days. (e) RAW264.7 cells were plated on the Osteo Assay Surface and were cultured with RANKL and M‐CSF for 6 days in the presence or absence of 200 ng/ml IL‐37b. Scale bar = 200 μm. Quantification of the bone resorption area on the Osteo Assay Surface. *N* = 4. The data are presented as the means ± SD. *p < 0.05, **p < 0.01. CCK‐8: cell counting kit‐8; IL‐37b: interleukin‐37b; M‐CSF: macrophage colony stimulating factor; RANKL: receptor activator of nuclear factor‐κB ligand; TRAP: tartrate‐resistant acid phosphatase [Color figure can be viewed at wileyonlinelibrary.com]

We performed the CCK‐8 assay to confirm that the actions of IL‐37b on osteoclast precursor RAW264.7 cells did not depend on cytotoxicity. On the basis of the cell proliferation data, treatment with 50–200 ng/ml IL‐37b for 48 or 72 hr was not toxic and did not reduce the number of cells (Figure 2a).

To observe the direct impact of IL‐37b on RANKL‐induced osteoclastogenesis, precursor cells were treated with M‐CSF and RANKL for 72 hr in the presence or absence of IL‐37b (50–200 ng/ml) and then stained with a TRAP staining. Notably, 200 ng/ml IL‐37b almost completely suppressed RANKL‐induced osteoclast formation and cells were replaced by TRAP‐negative cells (Figure 2b). The number of TRAP‐positive cells with three or more nuclei was reduced following the addition of IL‐37b (Figure 2c). Therefore, 200 ng/ml IL‐37b was used in subsequent experiments. Similarly, we also examined the effects of IL‐37b on osteoclast formation from bone marrow macrophages. After 5 days in culture, 200 ng/ml IL‐37b inhibited RANKL‐induced differentiation of  bone marrow derived macrophages (BMMs) into osteoclasts (Figure 2d). On the basis of our data, IL‐37b directly suppressed osteoclastogenesis in vitro.

To analyze the role of IL‐37b in osteoclastic bone resorption, we measured bone resorption in vitro and counted the number of bone lacunae. Precursor cells were seeded on bovine bone slides and were cultured on RANKL to produce osteoclasts. We performed the pit formation assay in 96‐well plates (Corning Osteo Assay Surface) and found that IL‐37b reduced RANKL‐mediated bone degradation. Meanwhile, RANKL‐induced osteoclasts formed large, deeper, and more extensive areas of bone degradation, whereas the addition of IL‐37b inhibited bone resorption (Figure 2E). Thus, IL‐37b negatively regulated osteoclast formation and bone resorption in vitro. Next, we selected RAW 264.7 cells as precursor cells for potential mechanistic studies.

### 3.4 IL‐37 inhibits osteoclast differentiation by downregulating the expression of NFATc1 in a MyD88‐dependent manner

The RANKL signaling pathway strongly activates NFATc1, the principal regulator of osteoclast differentiation (Lamoureux et al., 2014). As a ligand for RANKL, RANK activates the transcription factor complex AP‐1, which is composed of c‐Fos and c‐Jun (Asagiri & Takayanagi, 2007). In our study, we examined the effect of IL‐37b on the expression of the critical osteoclastogenic transcription factor NFATc1 to determine the molecular mechanism by which IL‐37b activity regulates osteoclastogenesis.

**Figure 3:**
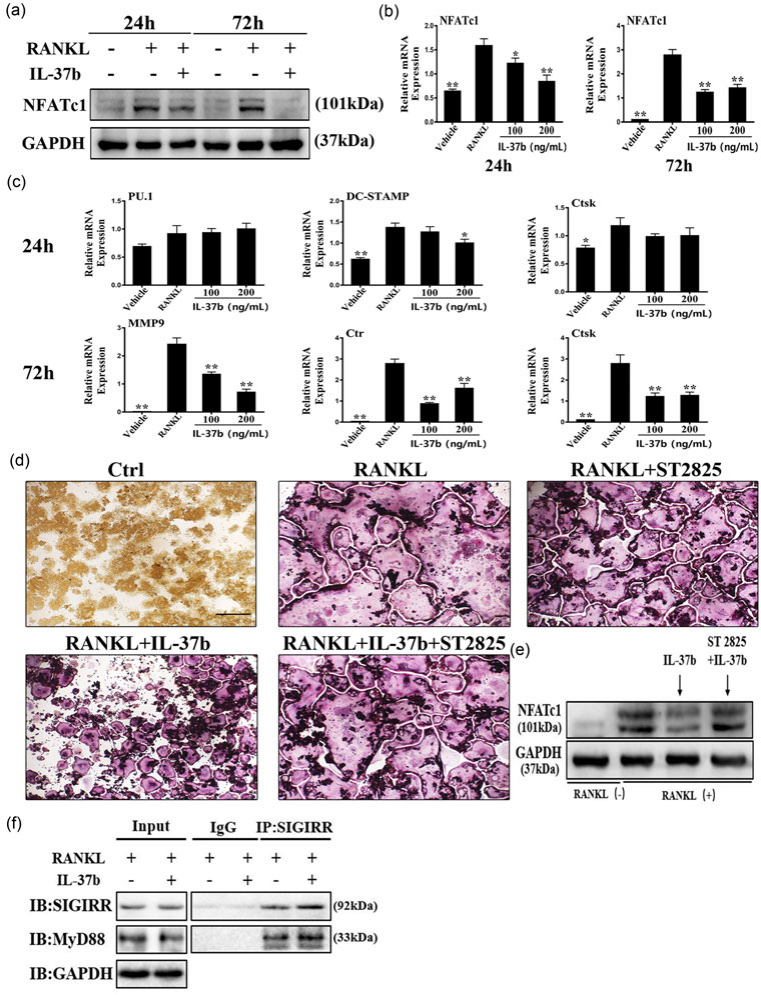
IL‐37b inhibited the expression of specific mRNAs and proteins at different stages of osteoclast differentiation. The MyD88 dimerization inhibitor (ST 2825) reversed the effects of IL‐37b on osteoclastogenesis. (a) Cells were treated with RANKL (50 ng/ml), M‐CSF (50 ng/ml), and IL‐37b (200 ng/ml) for 24 or 72 hr, and then lysed for Western blot analyses using antibodies against NFATc1 and GAPDH. (b,c) The expression of genes associated with osteoclast differentiation, fusion, and function were detected by qRT‐PCR. *p < 0.05 and **p < 0.01 compared with the RANKL‐treated control. (d) TRAP staining showed that 10 μg/ml ST 2825 partially counteracted the inhibitory effects of IL‐37b on osteoclast formation. Scale bar = 100 μm. (e) Western blot showing the level of NFATc1 in cells stimulated with RANKL in the presence or absence of ST 2825. The results were normalized to GAPDH levels. (f) Co‐IP analysis of the interaction between SIGIRR and MyD88 in RAW264.7 cells treated with RANKL and IL‐37b. Co‐IP: coimmunoprecipitation; GAPDH: glyceraldehyde 3‐phosphate dehydrogenase; IL‐37b: Interleukin‐37b; mRNA: messenger RNA; MyD88: myeloid differentiation factor 88; M‐CSF: macrophage colony stimulating factor; NFATc1: nuclear factor of activated T cells 1; qRT‐PCR: quantitative real‐time polymerase chain reaction; RANKL: receptor activator of nuclear factor‐κB ligand; SIGIRR: single Ig IL‐1‐related receptor; TRAP: tartrate‐resistant acid phosphatase [Color figure can be viewed at wileyonlinelibrary.com]

IL‐37b reduced the level of the NFATc1 protein level, as analyzed by Western blot analysis (Figure 3a). The qRT‐PCR analysis showed that IL‐37b attenuated RANKL‐induced expression of the NFATc1 mRNA during osteoclastogenesis (Figure 3b). In addition, the levels of mRNA associated with osteoclast formation were downregulated by the 24 or 72 hr IL‐37b treatment (Figure 3c). IL‐37b suppressed the expression of dendritic cell‐specific transmembrane protein, cathepsin K, MMP9, and calcitonin receptor. These genes are all closely related to osteoclast fusion and bone degradation (Dou et al., 2014). However, 100–200 ng/ml IL‐37b did not produce noticeable effects on the expression of the PU.1 mRNA compared with the untreated control group (Figure 3c). IL‐1 family members act directly or indirectly through MyD88 to transduce intracellular signals. Thus, the protein plays a critical role in the IL‐1 signaling pathway (Picard et al., 2003). ST 2825 is a specific inhibitor of MyD88, a small peptide compound that binds to the Toll/IL‐1 receptor (TIR) domain of MyD88. This compound inhibits MyD88 dimerization and prevents it from activating and transducing IL‐1 signaling, thereby blocking the IL‐1 signals (Loiarro et al., 2007). We examined whether IL‐37, a member of the IL‐1 family, also necessarily cooperate with the MyD88 protein in regulating osteoclast formation using the MyD88 inhibitor. The images of TRAP staining indicated that 10 μg/ml ST 2825 reversed the antiosteoclastogenic effects of IL‐37b (Figure 3d). Immunoblotting also showed that the inhibition of MyD88 contributed to the partial restoration of the of NFATc1 protein level (Figure 3e). Co‐IP revealed an interaction between SIGIRR and MyD88 when the precursor cells were treated with RANKL and IL‐37b (Figure 3f). SIGIRR is one of the important intracellular proteins involved in the IL‐37 anti‐inflammatory pathway (Nold‐Petry et al., 2015). On the basis of these results, IL‐37b negatively regulates osteoclast differentiation through the inhibition of NFATc1 expression in a MyD88‐dependent manner.

### 3.5 IL‐37 regulates osteoclast differentiation through the inhibition of NF‐κB signaling

**Figure 4:**
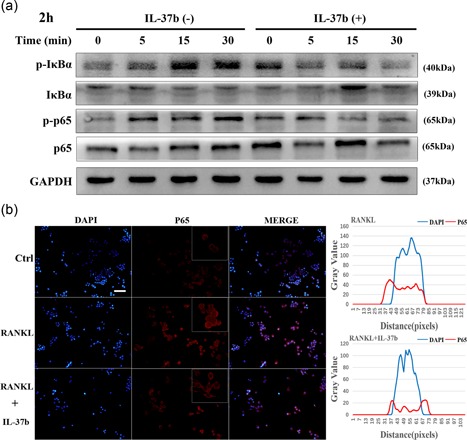
IL‐37b decreased the phosphorylation of p65 and IκB, intermediates in the classical NF‐κB pathway, in cells stimulated with RANKL, thereby reducing the translocation of the NF‐κB p65 subunit into the nucleus. (a) RAW264.7 cells were plated in six‐well plates and pretreated with or without IL‐37b (200 ng/ml) for 2 hr in the presence of M‐CSF (50 ng/ml) before RANKL stimulation for 0, 5, 15, and 30 min. Whole‐cell lysates were subjected to Western blot analyses with the indicated antibodies. GAPDH was used as the internal control. (b) RAW264.7 cells were seeded in 96‐well plates and treated with IL‐37b (200 ng/ml) for 2 hr, followed by stimulation with 50 ng/mll RANKL for 30 min. The intracellular location of the p65 subunit was observed by immunofluorescence staining. Five cells were selected from the RANKL group and the IL‐37b intervention group, respectively. The gray values of the red and blue staining were measured using the ImageJ software, and the mean values were plotted using excel. Scale bar = 200 μm. DAPI: 4’,6‐diamidino‐2‐phenylindole; GAPDH: glyceraldehyde 3‐phosphate dehydrogenase; IL‐37b: interleukin‐37b; M‐CSF: macrophage colony stimulating factor; NF‐κB: nuclear factor‐κB; RANKL: receptor activator of nuclear factor‐κB ligand [Color figure can be viewed at wileyonlinelibrary.com]

As shown in the current study, IL‐37 is involved in the mechanism regulating the expression of the essential transcription factor NFATc1 during osteoclast differentiation in a MyD88‐dependent manner. The activation of RANKL (RANK) in osteoclast precursor cells induces NFATc1 expression in the nucleus, an activity that is inseparable from the NF‐κB signaling pathway in this process. The classical NF‐κB pathway is required for osteoclastogenesis, and many target genes associated with osteoclast activation are also controlled by this signaling pathway (Boyle, Simonet, & Lacey, 2003). We investigated the activation of the canonical NF‐κB pathway after RANKL stimulation and further revealed the effect of IL‐37b on signal transduction during osteoclastogenesis. We observed the levels of phosphorylation IKB and p65 to avoid interference because the synthesis of the IKB protein is accompanied by an increase in the differentiation of monocytes into macrophages (Strålberg et al., 2017). Although the control group of cells stimulated with RANKL exhibited increased phosphorylation of IκBα and p65, IL‐37b significantly inhibited the activation of these proteins in response to the agonist (Figure 4a). NF‐κB proteins are stored in the cytoplasm of nonstimulated cells, and once cells are exposed to many stimuli, such as RANKL, the proteins are rapidly transported to the nucleus (Asagiri & Takayanagi, 2007). Therefore, we examined the subcellular localization of the NF‐κB p65 subunit in RAW264.7 cells using immunofluorescence staining. The nuclear localization of p65 was markedly increased in the cells treated with RANKL, but the nuclear translocation was suppressed upon the addition of IL‐37b. As shown in Figure 4b, five cells were randomly selected as the targets, and the fluorescence intensities of DAPI and p65 were measured. IL‐37b inhibited the entry of p65 into the nucleus. This finding was consistent with the result showing that the activity of the NF‐κB signaling pathway was increased in RANKL‐treated cells and decreased in the presence of IL‐37b.

### 3.6 IL‐37 negatively regulates LPS‐induced osteoclastogenesis

**Figure 5:**
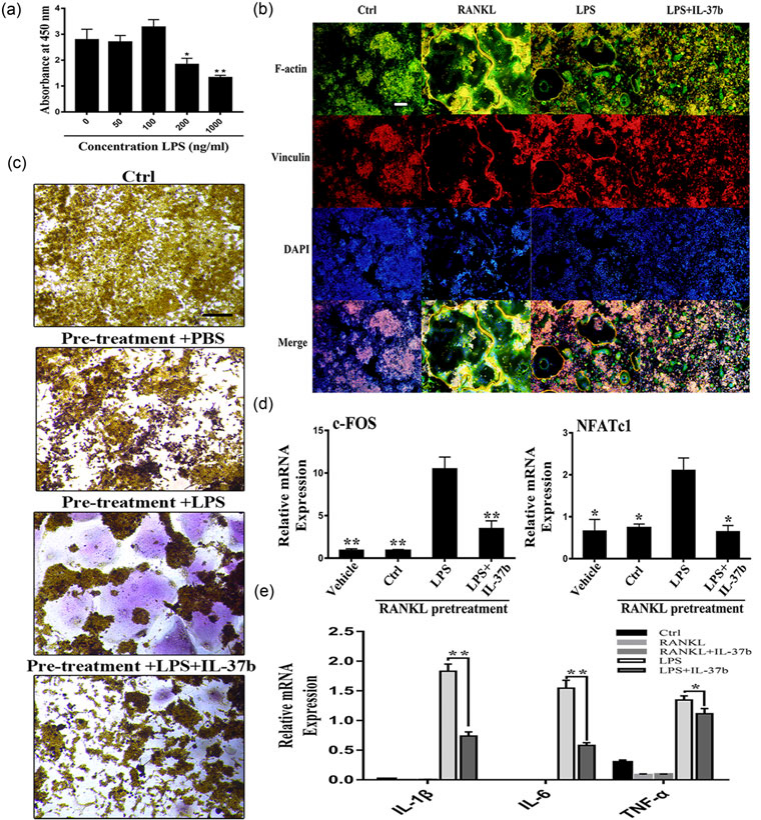
RAW264.7 cells that had been pretreated with RANKL for 24 hr were stimulated with LPS (100 ng/ml) to induce their differentiation into osteoclasts; IL‐37b reversed the process. (a) The CCK‐8 assay was used to examine the effect of LPS on progenitor cell proliferation. (b) IL‐37b inhibits RANKL or LPS‐mediated osteoclast fusion. A 24‐hr RANKL pretreatment triggered the fusion of precursor cells exposed to LPS conditions. Immunofluorescence staining revealed that IL‐37b reduced LPS‐induced actin ring formation. Scale bar = 200 μm. (c) After culture under the same induction conditions described above for three days, TRAP‐positive multinucleated cells were identified as osteoclasts. Scale bar = 200 μm. (d) RAW264.7 cells were treated with RANKL (50 ng/ml) for 24 hr and then stimulated with LPS (100 ng/ml) or LPS (100 ng/ml) and IL‐37b (200 ng/ml) for 24 hr. M‐CSF (50 ng/ml) was maintained in the culture media throughout the induction period. The relative expression levels of NFATc1 and c‐Fos were detected by quantitative PCR. (e) IL‐37b reduces the expression of proinflammatory cytokines during osteoclast differentiation. The levels of the IL‐1β, IL‐6, and TNF‐α mRNAs during the course of RANKL‐ or LPS‐induced osteoclastogenesis were analyzed by qRT‐PCR. *p < 0.05; **p < 0.01 compared with the LPS‐treated group. CCK‐8: cell counting kit‐8; IL‐37b: interleukin‐37b; LPS: lipopolysaccharide; M‐CSF: macrophage colony stimulating factor; NFATc1: nuclear factor of activated T cells 1; qRT‐PCR: quantitative real‐time polymerase chain reaction; RANKL: receptor activator of nuclear factor‐κB ligand; TNF: tumor necrosis factor; TRAP: tartrate‐resistant acid phosphatase [Color figure can be viewed at wileyonlinelibrary.com]

**Figure 6:**
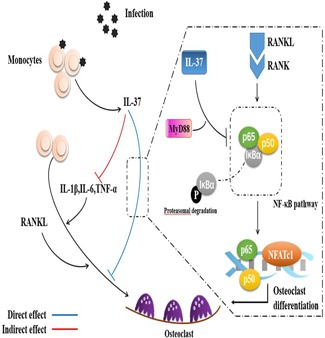
Interleukin 37 downregulates osteoclastogenesis through direct and indirect actions. IL: interleukin; MyD88: myeloid differentiation factor 88; NF‐kB: nuclear factor‐κB; RANKL: receptor activator of nuclear factor‐κB ligand; TNF‐α: tumor necrosis factor‐α [Color figure can be viewed at wileyonlinelibrary.com]

First, we determined the association between LPS and precursor cells in vitro. We examined the effect of LPS on the proliferation of precursor cells using the CCK‐8 assay (Figure 5a). LPS did not induce the formation of TRAP‐positive cells in the absence of pretreatment with RANKL, with or without M‐CSF (Supporting Information Figure 1A). In contrast, the addition of 100 ng/ml LPS significantly increased the apoptosis of precursor cells. Interestingly, progenitor cells pretreated with RANKL exhibited a reduced rate of apoptosis following stimulation with LPS (Supporting Information Figure 1B). By staining focal adhesions and the actin cytoskeleton, we observed the LPS‐mediated differentiation of precursor cells into osteoclasts, but both the size and number of nuclei formed were significantly less than cells stimulated with RANKL alone (Figure 5b). The results of TRAP staining also confirmed that LPS‐mediated osteoclastogenesis was not separable from the RANKL pretreatment (Figure 5c). Likewise, IL‐37b altered this process. Moreover, IL‐37b reduced the expression of the NFATc1 mRNA in precursor cells (Figure 5d). In fact, this culture was also used in some previous studies, because the production of osteoclasts is not completely avoidable as cells are exposed to RANKL in the microenvironment in vivo (Strålberg et al., 2017). In addition, we examined the expression of proinflammatory cytokines under physiological and pathological conditions because these factors have been shown to significantly promote osteoclast formation. Low levels of proinflammatory cytokines were detected following RANKL stimulation, further indicating that IL‐37b regulated the osteoclast differentiation through a mechanism that was not solely dependent on its anti‐inflammatory effects (Figure 5e). Therefore, the role of IL‐37b in LPS‐mediated osteoclastogenesis is at least mediated by the downregulation of inflammatory factors through the direct regulatory mechanism described above (Figure 6).

## 4 DISCUSSION

Bone regeneration and remodeling are processes involving the functions of osteoclasts, osteoblasts, and other related cells. In a complex microenvironment, the association between immune cells and bone‐related cells is essential for the repair and reconstruction of bones (Mountziaris, Spicer, Kasper, & Mikos, 2011). The occurrence of bone infections further strengthens the association between the immune system and the skeletal system. As shown in our previous study, the products of *Staphylococcus aureus*, such as staphylococcal protein A and staphylococcal lipoteichoic acid, affect the formation of osteoblasts and osteoclasts, changing the normal process of bone remodeling (Liu et al., 2017; Wang et al., 2017). In addition, bacteria cause bone necrosis not only by directly killing osteoblasts but also by the pathogen‐induced inflammatory response to osteoclastogenesis and bone degradation (Loi et al., 2016). Bone infection is an event in which inflammatory cells react to repair damaged tissues. Upon bone infection, macrophages secrete various cytokines and chemokines to recruit additional inﬂammatory cells, promote neovascularization, regulate mesenchymal stem cell (MSC) migration and differentiation, and mediate bone remodeling (Mountziaris et al., 2011). Of all the secreted factors, the most typical proteins include several cytokines, such as IL‐1β, IL‐6, and IL‐17; these cytokines promote bone resorption by enhancing osteoclast differentiation and activity or sometimes by inhibiting osteoblast differentiation, function, collagen synthesis, and bone formation (Cavagis et al., 2014). In contrast, the anti‐inﬂammatory cytokines IL‐10 and IL‐13 exert the opposite effect (Lorenzo, 2000).

IL‐37 is a recently discovered IL‐1 family member. It suppresses proinflammatory cytokine production, including IL‐1β, IL‐6, and IL‐17. However, it does not interfere with the production of anti‐inflammatory cytokines, including IL‐10. IL‐37 also inhibits dendritic cell activation and plays a role in adaptive immunity (Nold et al., 2010). Various normal tissues and diseased tissues express the different isoforms IL‐37 at various levels. Bone infection is a local manifestation of the systemic immune system and is accompanied by the differential expression of the cytokine. In our selected clinical specimens, significantly higher levels of IL‐37 were observed in infected sites than in the control sites, including the bone tissue and its surrounding soft tissue. Although the results showed a higher expression of IL‐37 in infected connective tissue, we considered the extent of the infection to be a more important factor. Previous studies have shown that IL‐37 is constitutively expressed in the cytoplasm of monocytes and dendritic cells (Li et al., 2015). Cytoplasmic IL‐37 is mainly located near the Golgi apparatus, endoplasmic reticulum, and plasma membrane, which also support its biological functions in the secretory pathway (Kumar et al., 2002). In addition, it was reported that monocytes have emerged as the major source of IL‐37 in human adult peripheral blood mononuclear cell (PBMCs), both basally and after LPS stimulation (Rudloff et al., 2017). Monocytes can differentiate into osteoclasts and secrete large amounts of IL‐37 when participating in inflammatory responses. Thus, a link may exist between IL‐37 and osteoclasts. We have shown that IL‐37 can indirectly affect the formation of osteoclasts by modulating the expression of proinflammatory cytokines, and we examined the direct action of IL‐37 on osteoclast precursor cells. In contrast, IL‐37 was previously shown to exert no direct effect on osteoclast formation in vitro, but the overall effect in vivo was positive (Saeed et al., 2016). Several explanations could account for the difference between the results, the first of which is the concentration of IL‐37. The action of IL‐37b (100 ng/ml) on osteoclastogenesis was limited, whereas an increase to 200 ng/ml IL‐37 resulted in a significant inhibition of osteoclast differentiation in vitro. Moreover, IL‐37b preserves the bone mass in both LPS‐injected and distal bone infection models. In conclusion, IL‐37 inhibits RANKL‐ and LPS‐induced osteoclast formation, as well as bone resorption, by inhibiting the expression of related cytokines and decreasing osteoclast formation from precursor cells.

NFATc1 is the master regulator of osteoclastogenesis and a common target gene of the essential transcription factors NF‐κB and c‐Fos (Danks & Takayanagi, 2013). It is essential for the expression of many osteoclast‐speciﬁc genes, such as cathepsin K (CtsK), TRAP, and calcitonin receptor. IL‐37 downregulated the expression of marker genes during OC differentiation and maturation, including the NFATc1, TRAP, and CtsK genes. This finding further supports the hypothesis that extracellular IL‐37 inhibits osteoclastogenesis in vitro. NF‐κB is a representative transcription factor that regulates inflammatory responses. In the skeletal system, the NF‐κB signaling pathway is directly involved in bone resorption‐related osteoclast differentiation and activation (Lin et al., 2017). The interaction between RANK and its ligands activates the canonical (p65/p50) and noncanonical (p52) NF‐κB pathways (Rho, Takami, & Choi, 2004). Various proinflammatory factors, such as TNF‐α, IL‐1β, and TLR ligands, promote osteoclast formation by activating the classical NF‐κB pathway in collaboration with RANKL signaling (Lin et al., 2017). As NF‐κB mediates both inflammation and osteoclastogenesis, it represents a potential therapeutic target for inflammatory bone diseases (Glass et al., 2011). NF‐κB activation has been inhibited using several methods, such as receptor inhibition, adaptor inhibition, IκB stabilization, and transcription factor inhibition (Rho et al., 2004). On the basis of our results, IL‐37 affected IκBα and NF‐κB (p65) activation in response to RANKL. However, IL‐37 also activated the NF‐κB pathway during the transient phase of monocyte‐macrophage differentiation. Further studies are needed to determine whether this process is related to the polarization of macrophages (Huang et al., 2015).

As infection‐induced bone destruction is primarily mediated by the host immune response and not simply by direct effects of pathogens; strategies designed to block cytokine production and RANKL modulators should be adjunct treatments to operations and antibiotic therapies (Mbalaviele, Novack, Schett, & Teitelbaum, 2017). Many cytokine inhibitors, including those targeting IL‐1β, IL‐6, and TNF‐α, have been approved for the treatment of inflammatory diseases, such as bone destruction and rheumatoid arthritis (Rao et al., 2013). Therapies with these antagonists have achieved good clinical outcomes, but have been limited by factors like stringent indications and more complications (Firestein & McInnes, 2017). Bone infection usually manifests as increased levels of multiple factors involved in the inflammatory response. Thus, for a certain cytokine, an antagonist treatment is often ineffective. IL‐37, a natural anti‐inflammatory factor, was shown to exert direct and indirect effects on bone resorption and osteoclast differentiation in our experiments while inhibiting the secretion of multiple inflammatory factors. In addition, angiogenesis and antibiotic treatments that reach the inflammatory sites are essential for bone healing. Some studies of ischemic injury have suggested that IL‐37 is involved in promoting angiogenesis through the TGF‐β pathway (Yang et al., 2015; Zhao et al., 2017). Further studies are needed to explain the impacts of these functions of IL‐37. Currently, two mechanisms have been identified to reveal the action of IL‐37. The first intracellular mechanism is based on SMAD3, which is a linker molecule downstream of the TGF‐β signaling pathway. The second mechanism relies on the formation of the trimeric complex of IL‐37, SIGIRR, and IL‐18Rα on the cell surface (Nold‐Petry et al., 2015). MyD88 and SIGIRR interact with each other in the HEK293 cell line following TLR4 activation (Qin, Qian, Yao, Grace, & Li, 2005). After stimulation of the intrinsic model, SIGIRR interacts with MyD88 and suppresses the innate immune response. However, further investigations of the mechanism by which intracellular SIGIRR interacts with MyD88, a TLR adapter, when the inflammatory reaction is triggered are needed (Zhang, Wu, Zhao, Deng, & Qian, 2011). Similarly, our data also suggest that other auxiliary proteins, such as MyD88, may be recruited in stimulated precursor cells, leading to the inhibition of osteoclastogenesis. Although immunoprecipitation experiments confirmed the interaction between MyD88 and SIGIRR in our study, further studies are required to determine the explanation for the increased expression of MyD88 in the IL‐37 treatment group. At the same time, other issues need to be addressed; for example, the suppressed immune response appears to be accompanied by a restriction of the host antibacterial mechanism. In a mouse model of infection with *Streptococcus pneumoniae*, the data showed that an initial intervention with IL‐37 was detrimental to the prevention of *S. pneumoniae* infection, which promoted the bacteremic spread and increased mortality (Schauer et al., 2017). Thus, the appropriate timing and dosage of the treatment of IL‐37 are critical to alleviate inflammatory bone destruction and reduce the risk of systemic infection.

In summary, although the expression of endogenous IL‐37 in patients with bone infection is not sufficient to induce an uncontrolled inflammatory response, the pathological process may be reversed by the exogenous administration of this cytokine. Therefore, based on accumulating evidence, the application of IL‐37 as an immunomodulatory therapy may become a new bone infection treatment.

## CONFLICTS OF INTEREST

The authors declare that there are no conflicts of interest.

## AUTHOR CONTRIBUTIONS

R. T., S. D., and J. F. contributed to experimental design. R. T., J. Y., W. L., and Y. C. contributed to the acquisition of experimental data. R. T., J. Y., J. F. jointly completed the collection of clinical specimens. R. T. and J. Y. were responsible for statistical analysis. R. T., S. D., and J. F. completed the manuscript. All authors approved the version of the manuscript.

## Supporting information

Supporting Information

Supporting Information
