# Supplementary figures and images for: Interleukin‐37 inhibits osteoclastogenesis and alleviates inflammatory bone destruction
Source: J Cell Physiol. 2018 Nov 10;234(5):7645–58. doi: 10.1002/jcp.27526 (PMC6587950; doi:10.1002/jcp.27526)

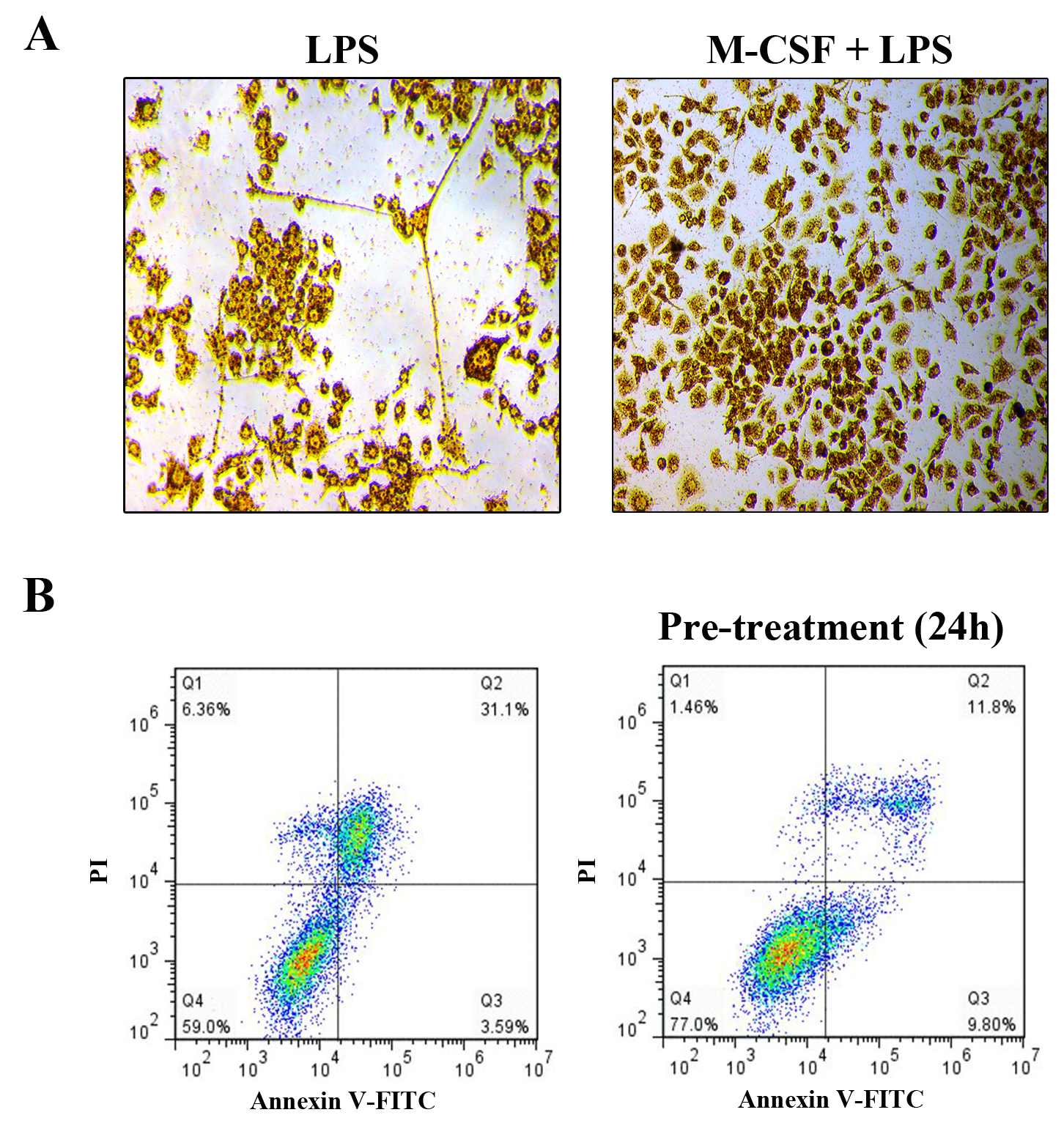

Supplement: Supplementary file 1 — Supporting Information [file JCP-234-7645-s001.tif]
