# Supplementary material for: Interleukin‐37 inhibits osteoclastogenesis and alleviates inflammatory bone destruction
Source: J Cell Physiol. 2018 Nov 10;234(5):7645–58. doi: 10.1002/jcp.27526 (PMC6587950; doi:10.1002/jcp.27526)
Supplement: Supplementary file 2 — Supporting Information [file JCP-234-7645-s002.docx]

Supplementary Figure 1. (A) RAW264.7 cells were stimulated with LPS (100▒ng/ml) for 3 days in the presence or absence of M-CSF (50▒ng/ml). TRAP-positive cells were not present in all images. **(B)** The cells in the RANKL pretreatment group and the control group were treated with LPS (100▒ng/ml) for 24▒hours and stained with Annexin-V-FITC and PI for 15▒minutes to detect apoptosis. The RANKL pretreatment increased cell survival following LPS stimulation.
